# Targeted deep sequencing of mucinous ovarian tumors reveals multiple overlapping RAS-pathway activating mutations in borderline and cancerous neoplasms

**DOI:** 10.1186/s12885-015-1421-8

**Published:** 2015-05-19

**Authors:** Robertson Mackenzie, Stefan Kommoss, Boris J. Winterhoff, Benjamin R. Kipp, Joaquin J. Garcia, Jesse Voss, Kevin Halling, Anthony Karnezis, Janine Senz, Winnie Yang, Elena-Sophie Prigge, Miriam Reuschenbach, Magnus Von Knebel Doeberitz, Blake C. Gilks, David G. Huntsman, Jamie Bakkum-Gamez, Jessica N. McAlpine, Michael S. Anglesio

**Affiliations:** 1 Molecular Oncology, BC Cancer Agency Research Centre, Vancouver, Canada; 2 Pathology and Laboratory Medicine, University of British Columbia, Vancouver, Canada; 3 Gynecology and Obstetrics, Tuebingen University Hospital, Tuebingen, Germany; 4 Laboratory Medicine and Pathology, Mayo Clinic, Rochester, USA; 5 Applied Tumor Biology, Institute of Pathology, University of Heidelberg, Heidelberg, Germany; 6 Gynecology and Obstetrics, Division of Gynecologic Oncology, University of British Columbia, Vancouver, Canada; 7 Gynecology and Obstetrics, Mayo Clinic, Rochester, USA; 8 Department of Pathology and Laboratory Medicine, University of British Columbia, Vancouver, Canada

**Keywords:** Next-generation sequencing, Mucinous, Ovarian, BRAF, KRAS, TP53, Heterogeneity

## Abstract

**Background:**

Mucinous ovarian tumors represent a distinct histotype of epithelial ovarian cancer. The rarest (2-4 % of ovarian carcinomas) of the five major histotypes, their genomic landscape remains poorly described. We undertook hotspot sequencing of 50 genes commonly mutated in human cancer across 69 mucinous ovarian tumors. Our goals were to establish the overall frequency of cancer-hotspot mutations across a large cohort, especially those tumors previously thought to be “RAS-pathway alteration negative”, using highly-sensitive next-generation sequencing as well as further explore a small number of cases with apparent heterogeneity in RAS-pathway activating alterations.

**Methods:**

Using the Ion Torrent PGM platform, we performed next generation sequencing analysis using the v2 Cancer Hotspot Panel. Regions of disparate ERBB2-amplification status were sequenced independently for two mucinous carcinoma (MC) cases, previously established as showing ERBB2 amplification/overexpression heterogeneity, to assess the hypothesis of subclonal populations containing either *KRAS* mutation or *ERBB2* amplification independently or simultaneously.

**Results:**

We detected mutations in *KRAS*, *TP53*, *CDKN2A*, *PIK3CA*, *PTEN*, *BRAF*, *FGFR2*, *STK11*, *CTNNB1*, *SRC*, *SMAD4*, *GNA11* and *ERBB2. KRAS* mutations remain the most frequently observed alteration among MC (64.9 %) and mucinous borderline tumors (MBOT) (92.3 %). *TP53* mutation occurred more frequently in carcinomas than borderline tumors (56.8 % and 11.5 %, respectively), and combined IHC and mutation data suggest alterations occur in approximately 68 % of MC and as many as 20 % of MBOT. Proven and potential RAS-pathway activating changes were observed in all but one MC. Concurrent *ERBB2* amplification and *KRAS* mutation were observed in a substantial number of cases (7/63 total), as was co-occurrence of *KRAS* and *BRAF* mutations (one case). Microdissection of *ERBB2*-amplified regions of tumors harboring *KRAS* mutation suggests these alterations are occurring in the same cell populations, while consistency of *KRAS* allelic frequency in both *ERBB2* amplified and non-amplified regions suggests this mutation occurred in advance of the amplification event.

**Conclusions:**

Overall, the prevalence of RAS-alteration and striking co-occurrence of pathway “double-hits” supports a critical role for tumor progression in this ovarian malignancy. Given the spectrum of RAS-activating mutations, it is clear that targeting this pathway may be a viable therapeutic option for patients with recurrent or advanced stage mucinous ovarian carcinoma, however caution should be exercised in selecting one or more personalized therapeutics given the frequency of non-redundant RAS-activating alterations.

**Electronic supplementary material:**

The online version of this article (doi:10.1186/s12885-015-1421-8) contains supplementary material, which is available to authorized users.

## Background

Mucinous ovarian tumors are a rare histological type of epithelial ovarian cancer (EOC), representing 2-4 % of these malignancies [1–4]. Primary mucinous ovarian carcinomas are distinct from other EOC in both presentation and outcome [3, 5–8]. Believed to develop along a continuum from benign cysts to borderline tumors to invasive carcinomas, the majority of cases present as borderline tumors (MBOT) or stage I mucinous carcinomas (MC). Overall, prognosis is excellent, although in rare cases where cancer has spread beyond the ovaries, outcomes and response to conventional chemotherapy is poor.

In addition to sharing many biomarkers, MCs are morphologically similar to adenocarcinomas of the pancreas and gastrointestinal tract, posing a challenge in differentiating primary ovarian tumors from metastatic disease [9–11]. Given the number of shared features between these disease entities, including a dominance of RAS-activating changes, there is a potential for similar therapeutic strategies and “umbrella” trials in women with advanced stage or recurrent disease [12, 13].

Among mucinous tumors, the most prevalent mutations occur in the mitogen activated protein kinase (MAPK) pathway, including *KRAS* mutations and ERBB2 amplification/overexpression [13]. Historically, *KRAS* mutations have been observed in greater than 75 % of mucinous ovarian tumors, although differentiation of MBOT from MC and exclusion of metastatic disease have not consistently been applied in studies of this disease type [14–16]. Copy number analyses have implicated loss of heterozygosity of chromosomal regions 9p, 17p and 21q in the potential development of these tumors [17]. Additional mutations have been observed in *BRAF*, *TP53*, *PTEN*, *PIK3CA* and more recently *CDKN2A* and *RNF43* [14, 18–20]. However, rarity of the disease has limited large-scale analyses of mutational frequency among mucinous ovarian tumors [19, 21]. Furthermore, apparent intratumoral heterogeneity among mucinous tumors represents an interesting challenge for molecular profiling and potential personalized therapeutic strategies [13, 22].

**Fig. 1:**
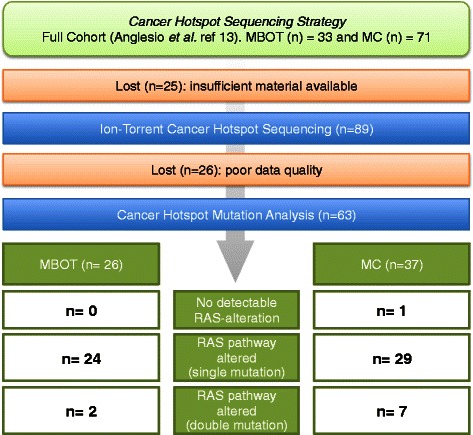
Outline of next-generation sequencing based sequencing strategy in the context of previously established cohort RAS-alterations defined in Anglesio et al., 2013 [13]. Direct RAS-pathway alterations including suspected and known activating alteration to KRAS, BRAF, ERBB2, FGFR2, and STK11 (the latter is presumed to alleviate negative signals on mTOR via TSC1/2 complex, similar to the effect of ERK1/2 activation)

Our group recently reported on the most frequently observed molecular alterations across mucinous tumors, observing *KRAS* mutations in 43.6 % MCs and 78.8 % MBOTs and ERBB2 amplification/overexpression in 18.8 % MCs and 6.2 % MBOTs, the latter being assessed by immunohistochemistry, fluorescent- and chromogenic-*in situ* hybridization (IHC, FISH & CISH) [13]. This analysis suggested tumors lacking ERBB2 or *KRAS* abnormalities tend to have poor prognosis, raising the question of whether an alternative mutation may be contributing to the pathology of this group [13]. In the current study, we applied targeted deep sequencing to the same cohort from our previous study [13], acquiring data for 37 MC and 26 MBOT. Two primary goals were sought: first, to search for molecular alterations that may contribute to the pathogenesis of mucinous tumors without apparent RAS-activating alterations and second, to investigate heterogeneity observed in seemingly rare RAS-pathway “double-hit” cases discovered in our previous study [13]. An outline of our sequencing strategy and resultant data is given in Fig. 1.

## Methods

### Sample cohort

Collection of specimens for experimental analysis was performed by the OVCARE tumor bank and the Mayo Clinic, use of material was approved by the UBC-BCCA Research ethics board. All specimens underwent review of pathology reports (authors CBG, JNM) as well as single slide review of sampled material (author ANK and MSA) to confirm diagnosis and establish cellularity. Assessment of HPV infection was performed [23] to rule out the possibility of rare metastasis from the cervix presenting with mucinous histology in the ovary and all cases were negative. DNA was extracted from formalin fixed paraffin embedded (FFPE) tissue for sequencing analysis. Where noted, microdissection of potentially distinct cell populations was performed using ERBB2-IHC stained sections as a guide.

### Ion torrent sequencing

Although we attempted to include the entire cohort described in our previous study [13], we were limited by availability and quality of material. DNA isolated from FFPE tissue was available for 89 mucinous tumors, including 30 MBOT and 59 MC. Following quality control processing (described below), 37 MC and 26 MBOT remained. Amplicon libraries were prepared and barcoded using the commercially available Cancer Hotspot Panel v2 primer pool and IonXpress barcode adapter kit as previously described [24, 25]. Libraries were quantified using Agilent High Sensitivity DNA chips, 20pM barcoded libraries were pooled (4 samples at a time), clonally amplified onto IonSphere particles using the Ion OneTouch system, and loaded on Ion 316 chips for sequencing. Variant calling was performed using the Ion Torrent Variant Caller with hg19 as a reference.

### Data processing and quality review

Successful sequencing was defined when there was at least 100x average depth of coverage for >80 % of amplicons sequenced. Individual cases were manually reviewed to evaluate overall sequencing quality (e.g., the number of variant calls due to sequencing artifacts [26], percentage of reads mapping to target region, etc.). Cases with poor quality (n = 26) on manual review were excluded. We report only on variants observed at >5 % allelic frequency and >10x coverage, that correspond to non-synonymous changes occurring in “hostspot” regions previously reported to be somatic in COSMIC (Catalogue of somatic mutations in cancer) [27], or are otherwise presumed to be deleterious and somatic if the given point mutation or insertion/deletion resulted in early termination.

### Immunohistochemistry

ERBB2 IHC (scored according to ASCO/CAP guidelines [28]) was performed exactly as described in previously [13]. An ERBB2 IHC score of 3+ was used as a proxy for amplification status as this has been previously shown to be highly concordant in these and other tumor types (e.g. breast) [13, 29]. IHC for p53 was generated as described previously [30] and scored on the same 3-tier system: 0 = complete absence, 1 = up to 50 % nuclear positivity and 2 = greater than 50 % nuclear positivity. IHC for p53 was considered a proxy for mutations, where both the null phenotype (0) and strongly positive (2) were considered abnormal [30].

## Results

### Ion torrent sequencing

**Fig. 2:**
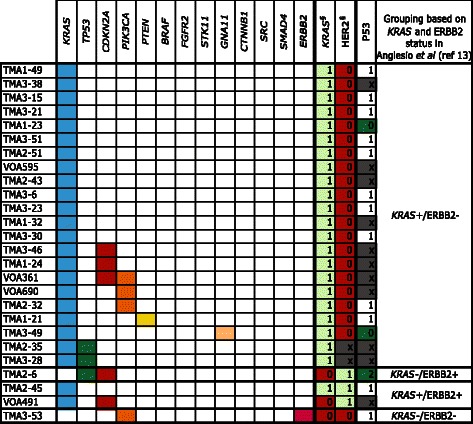
Mutation frequencies and immunohistochemistry scores for 26 mucinous borderline tumors. Solid color in any of the first 13 columns represents a presumed somatic (COSMIC) hotspot mutation in the given case. In the last three columns numbers represent binarized IHC score for p53 and ^§^ “Original ERBB2 amplification and *KRAS* mutation” status derived from Anglesio et al., 2013 [13] where 0 = Negative, 1 = Positive, X = Unknown, the latter derived from IHC, FISH, and/or CISH. IHC for p53 is displayed as three-tiered IHC score where 0 (no staining) and 2 (>50 % positive nuclei) represent abnormal p53 status and 1 (1-50 % positive nuclei) represents normal p53 status (x = data unavailable)

**Fig. 3:**
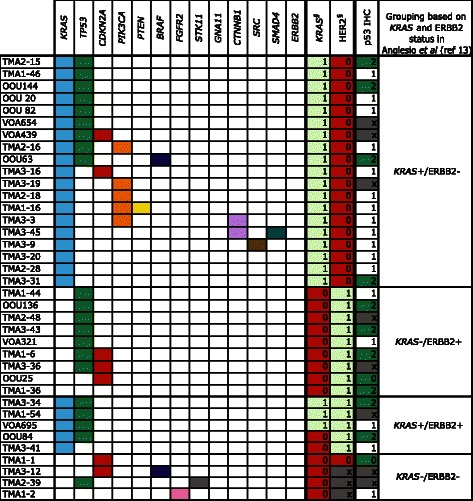
Mutation frequencies and immunohistochemistry scores for 37 mucinous carcinoma. As in Fig. 2, Solid color in any of the first 13 columns represents a presumed somatic (COSMIC) hotspot mutation in the given case. In the last three columns numbers represent binarized IHC score for p53 and ^§^ “Original ERBB2 amplification and *KRAS* mutation” status derived from Anglesio et al., 2013 [13] where 0 = Negative, 1 = Positive, X = Unknown, the latter derived from IHC, FISH, and/or CISH. IHC for p53 is displayed as three-tiered IHC score where 0 (no staining) and 2 (>50 % positive nuclei) represent abnormal p53 status and 1 (1-50 % positive nuclei) represents normal p53 status

Quality sequencing data was obtained for 63 cases of primary ovarian mucinous tumors including 26 borderline and 37 carcinomas (Fig. 1). Deleterious somatic mutations were observed within 13 genes: *KRAS*, *TP53*, *CDKN2A*, *PIK3CA*, *PTEN*, *BRAF*, *FGFR2*, *STK11*, *CTNNB1*, *SRC*, *SMAD4*, *GNA11*, and *ERBB2* (Table 1). Ion Torrent sequencing validated previously observed Sanger results for *KRAS* mutations [13] and identified three additional *KRAS* variants that were not detectable by Sanger (likely due to low cellularity and restriction of the previous study to the amino acid 12/13 hotspot region. (Figs. 2 & 3; Additional file 1). Additional variants were found in one MBOT and two MC: MBOT: VOA491 - p.Gly12Val; MC: OOU84 - p. Ala59Gly; and TMA3-41 - p.Gly12Val.

**Table 1:** Somatic hotspot mutation frequencies for MC and MBOT

Carcinoma (n = 37)	Mutation Events		Frequency
*KRAS*	24		64.9
*TP53*	24	*	56.8
*CDKN2A*	8	*	18.9
*PIK3CA*	5		13.5
*PTEN*	2	*	2.7
*BRAF*	2		5.4
*FGFR2*	1		2.7
*STK11*	1		2.7
*CTNNB1*	2		5.4
*SRC*	1		2.7
*SMAD4*	1		2.7
Total Number Mutations	71		
*ERBB2 Amplification*	*14*	^*§*^	*37.8*
Borderline Tumor (n = 26)	Mutation Events		Frequency
*KRAS*	24		92.3
*TP53*	3		11.5
*CDKN2A*	5		19.2
*PIK3CA*	4		15.4
*PTEN*	1		3.8
*GNA11*	1		3.8
*ERBB2*	1		3.8
Total Number Mutations	39		
*ERBB2 Amplification*	*3*	^*§*^	*11.5*
*Total # Mutations*	*110*		

*Multiple cases with 2 mutation events. Number of mutated cases were used to establish frequency across cohort: *TP53* (n = 21), *CDKN2A* (n = 7) and *PTEN* (n = 1)

^§^Derived from Anglesio et al., 2013 [13]

### Mucinous borderline tumors

Among 26 MBOT cases, 39 presumed somatic mutations were detected across seven genes: *KRAS* (24/26; 92.3 %), *TP53* (3/26; 11.5 %), *CDKN2A* (5/26; 19.2 %), *PIK3CA* (4/26; 15.4 %), *PTEN* (1/26; 3.8 %), *GNA11* (1/26; 3.8 %), and *ERBB2* (1/26; 3.8 %) (Table 1 & Fig. 2). Amongst these MBOTs, *KRAS* mutations involved the “hotspot” for Gly-12 only (Additional file 1). When grouped based on *KRAS* hotspot mutant and ERBB2 amplification status we observed 22 (84.6 %) *KRAS*+/ERBB2-, one (3.8 %) *KRAS*-/ERBB2+, two (7.7 %) *KRAS*+/ERBB2+, and one (3.8 %) *KRAS*-/ERBB2-; however, this last case harboured an *ERBB2* p.Asp769Asn mutation rather than amplification. Despite the moderate frequency of amplification events, activating mutations of *ERBB2* have not previously been implicated in mucinous carcinoma pathogenesis. Mutations to the 769 residue are expected to have an activating effect given they are within the protein kinase domain [31–33]. Such mutations have been reported previously in both lung and esophageal cancers [34, 35].

### Mucinous carcinoma

Within our cohort of 37 MC, we found 71 presumed somatic mutations within 11 different genes: *KRAS* (24/37; 64.9 %), *TP53* (21/37; 56.8 %), *CDKN2A* (7/37; 18.9 %), *PIK3CA* (5/37; 13.5 %), *PTEN* (1/37; 2.7 %), *BRAF* (2/37; 5.4 %), *FGFR2* (1/37; 2.7 %), *STK11* (1/37; 2.7 %), *CTNNB1* (2/37; 5.4 %), *SRC* (1/37, 2.7 %), and *SMAD4* (1/37; 2.7 %) (Table 1 & Fig. 3). Three cases had two different, non-synonymous mutations in *TP53* (OOU20, VOA439, TMA1-6), one case had two mutations observed in *CDKN2A* (OOU25), and one case had two *PTEN* mutations (TMA1-16). With a single exception (OOU84 - p.Ala59Gly), *KRAS* mutations involved the Gly-12 residue. Co-occurrence of multiple mutations (including double hits to the RAS-pathway) was observed at a higher frequency within MC (26/37; 70.3 %) over MBOT (12/26, 46.2 %), however was not statistically significant (Fisher exact test p = 0.0634).

Grouping of MC based on ERBB2 and *KRAS* status resulted in 19 (51.4 %) *KRAS*+/ERBB2-, nine (24.3 %) *KRAS*-/ERBB2+, five (13.5 %) *KRAS*+/ERBB2+, one (2.7 %) *KRAS*-/ERBB2-, and three KRAS- cases undefined *ERBB2* amplification status (Fig. 3). Among the three *KRAS*-/ERBB2 undefined cases, alternative RAS-pathway activating mutations were observed in two cases (TMA1-2: *FGFR2* p.Ser252Trp; TMA3-12: *BRAF* p.Val600Glu), and the third (TMA2-39) having an *STK11* inactivating change that may result in alleviation of negative signals on mTOR via TSC1/2 complex, similar to the effect of ERK1/2 activation [36, 37]. Ultimately, one case (TMA1-1) is definitively negative with respect to RAS-alteration status given the current screen.

### TP53 status amongst mucinous tumors

Immunohistochemical scoring of p53 expression was generally concordant with mutation status (Figs. 2 & 3). A TMA-based evaluation of p53 protein was done for the full cohort of Mayo and Vancouver samples, with interpretable results obtained for 15/26 MBOT and 29/37 MC where sequencing was also available. Of these, three MBOT cases had abnormal staining patterns for p53, and occurred in *KRAS* mutant or *ERBB2* amplified cases. TMA1-23 and TMA3-49 showed complete loss of p53 staining; however, no mutation was observed in the regions sequenced, which may be the result of larger deletions or mutation outside of the hotspot panel. Twelve MC cases had abnormal p53 staining and appeared to be well distributed across all four groups of *KRAS* mutant and *ERBB2* amplified groups. Four cases (TMA3-31, OOU25, TMA1-36 and TMA1-1) had p53 staining abnormalities that occurred without detectable mutation. Finally, seven MC (18.9 %; TMA1-46, OOU 20, OOU 82, TMA2-16, TMA1-44, VOA 321 and VOA 695) were found to have presumed-somatic *TP53* mutations, but did not have corresponding IHC abnormalities. It should be noted that the Ion-Torrent panel does not sequence the entirety of *TP53* and is not well suited for the detection of exon-level (or larger) deletions, which may result in a null-phenotype by IHC. Further, our analysis may be partially confounded by non-somatic variants, whether contaminating the COSMIC database (“false-positive”, non-somatic in our context), or having subtle effects on protein stability/unknown functional effects: i.e. the presence of a “presumed somatic mutation” may not yield a mutant overexpression or null-phenotype. Overall, *TP53* mutations were more prevalent in MC, and no enrichment of *TP53* was associated with any RAS-pathway mutation groups. Using p53 IHC data alone (Additional file 2) and expanding to all available cases, we observed no difference in overall or progression-free survival for the MC cohort (Additional file 3). Corresponding survival analysis for borderline tumors was uninformative due to cohort sample size and censoring. Our data set also failed to show enrichment of *TP53* mutation, in either borderline or carcinomas.

### RAS-pathway heterogeneity

**Fig. 4:**
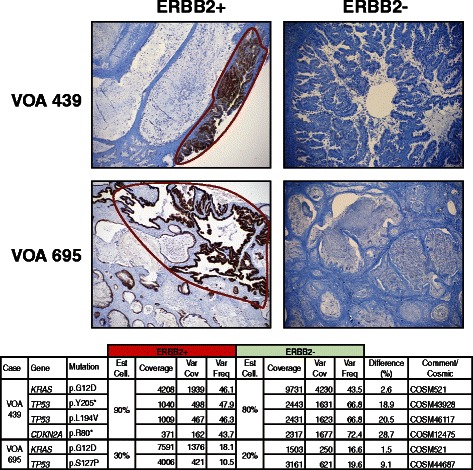
ERBB2 immunohistochemical heterogeneity in two MC and sequencing results from each distinct component. ERBB2+ regions were microdissected and sequenced independently from the ERBB2- components to compare mutation events. Identical *KRAS* mutations were observed in the ERBB2+ and ERBB2- regions for both cases. ERBB2 high-intensity staining regions was used as a proxy for gene amplification status, as regions previously defined by this high-level IHC staining correlated perfectly with FISH and/or CISH data suggesting amplification of the *ERBB2* gene [13]

Two cases of MC (VOA695 and VOA439) were previously described to be heterogeneous for ERBB2 amplification/overexpression [13]. As greater access was available for these local cases, a full series of clinical blocks was examined for ERBB2 3+ and negative IHC. Positive and negative regions were then fine-needle microdissected with both front and back ERBB2-stained sections as a guide to ensure consistency in IHC positive (3+) and negative (0) regions. Sequencing of the disparate regions of VOA439 confirmed the previously observed *KRAS* p.Gly12Asp mutation at similar allelic frequency in both ERBB2+ and ERBB2- regions: 46.1 % and 43.5 % respectively (Fig. 4). Two *TP53* and one *CDKN2A* mutations were also found in both regions at similar allelic frequencies. Similar results were observed in case VOA695 across ERBB2+ and ERBB2- regions: *KRAS* p.Gly12Asp mutation at 18.1 % and 16.6 %, and *TP53* p. Ser127Pro mutation at 10.5 % and 19.6 % allelic frequency, respectively. Double-hit RAS-pathway alterations were confirmed in six additional MC cases (total 21.6 %). Double-hits were observed in both MBOT (two cases; 7.7 %) and MC, but were more prevalent in MC. In general, examination of allelic ratios of RAS-pathway alterations in comparison to cellularity estimates suggested that RAS-pathway mutations may be more likely to be hemizygous or homozygous (Additional file 1) although copy number analysis was not available to validate this.

## Discussion

In the current study we provide quantitative interrogation of MC and MBOTs using amplicon-based hotspot sequencing. Our re-sequencing efforts confirmed *KRAS* mutations to be the most frequent molecular alteration amongst mucinous tumors, appearing more common in borderline malignancies over carcinomas (92.3 % versus 64.9 %, respectively; Fisher exact p = 0.0157). These values reflect what was previously reported [13]; however, improved sensitivity through the use of next generation sequencing identified *KRAS* mutations in three cases previously believed to be wild type (one MBOT and two MC). We further added to the complement of known RAS-activating mutations in observing mutations in *BRAF* (two MC), as well as previously unreported potentially RAS-activating alterations in *FGFR2*, *ERBB2*, and *STK11*, each affecting a single carcinoma. As noted above, inactivating mutation of *STK11* could be considered an alternative mechanism to RAS-activation outside of typical *KRAS/BRAF* mutations [36, 37], an important point given the occurrence of this mutation in one of only two MC without other known RAS alterations. Most other mutations observed here have previously been implicated in the biology of mucinous ovarian tumors (*KRAS*, *BRAF*, *TP53*, *CDKN2A*, *PIK3CA*, *PTEN*) [14, 15, 18–20, 38]. Reported mutation frequencies vary, with small sample size and inconsistent diagnostic criteria likely at the heart of the variance observed in the literature. To the best of our knowledge, mutations within *FGFR2*, *ERBB2* (missense/activating), *STK11*, *GNA11*, *SRC*, *CTNNB1*, and *SMAD4* have not been previously reported in mucinous ovarian tumors. *GNA11* mutations, such as the one observed in an MBOT have been shown to up-regulate RAS-pathway activation [39], and while *SRC* mutations have not been previously reported in ovarian MC, others have suggested a high level of SRC protein kinase activity in these tumors [40, 41].

Amongst our cohort only one MC remained without identifiable RAS-pathway alteration, all but eradicating the RAS-activation negative group. Although relatively broad, our screen was not genome-wide and it is foreseeable that other rare RAS-activating alterations could be uncovered. This re-analysis also implies there is little difference in survival in tumors lacking RAS-pathway alterations, if any of these so-called “RAS-negative” tumors exist. We were also unable to show survival difference between ERBB2-positive, *KRAS*-positive, or non-*KRAS*/ERBB2-altered cases. However, it should be noted that our total cohort numbers have depleted since our previous analysis, and with additional RAS-pathway alterations defining unique groups, the number of samples per group were insufficient for meaningful conclusions on outcome.

Intratumoral heterogeneity among mucinous ovarian tumors, which previously seemed to be restricted to heterogeneity in ERBB2 status (observable *in situ* using FISH, CISH or IHC), presents a challenge for standard molecular analyses [13]. Based on our previous data suggesting a near-mutual exclusivity of RAS-pathway alterations in MC as well as numerous similar examples in the literature [42–44], we expected *KRAS* mutations would be restricted to regions lacking ERBB2 positivity. Surprisingly, *KRAS* mutations were found at near-identical frequencies in both ERBB2+ and ERBB2- regions of both examined MC. In fact, multiple alterations to the RAS-pathway were observed within two MBOT and six MC. This suggests that the *KRAS* mutations in both of these cases represent an ancestral alteration, present prior to the amplification of *ERBB2.* Further, this supports a model wherein RAS-pathway alterations are unlikely to be functionally equivalent.

Alterations involving the *TP53* locus occurred more frequently in MC than MBOT (21/37; 56.8 % and 3/26; 11.5 %, respectively). Aberrant expression of p53, assessed by IHC (scores of 0 and 2), suggest underlying genetic alterations in cases where no mutation were observed, a distinct possibility given the limits of our screening strategy. Considering both IHC and sequencing data, we estimate the frequency of TP53 alterations to be slightly higher than indicated in the mutation data alone and we estimate rates of approximately 20 % and 68 % for MBOT and MC, respectively. Unfortunately, we were unable to show an effect for p53 mutation (based on IHC status or mutation status) on patient outcomes in either MBOT or MC (Additional file 3). It may be reasonable to suggest acquisition of *TP53* mutation imparts genomic instability that in turn leads to accumulation of other mutations permissive overcoming senescence and other anti-growth signals induced by constitutive RAS-activation (for example through acquisition of PTEN loss of function mutations seen here). Should a suitable cohort be identified, a future study may be able to evaluate accumulated DNA copy number changes and clonal composition between MBOT and MUC. This may suggest a correlation between genomic complexity and acquisition of p53 mutations and/or secondary RAS-activating mutations, however this is conjecture at this point.

## Conclusions

Previous data on mucinous ovarian cancers suggested a less favorable prognosis for cases not carrying a known RAS-pathway alteration [13], similar to reports in the ovarian low-grade serous/serous borderline tumor spectrum [45]. However, this finding is not reproducible in our current study where greater sensitivity in detection is applied and additional RAS-pathway alterations are considered. In general, we saw an increased frequency of multiple RAS-pathway alterations and *TP53* mutations amongst carcinomas versus borderline tumors in our cohort, suggesting mutations in both of these pathways are critical in accelerating the progression of mucinous ovarian tumors. Save for a single case of MC, RAS-pathway activation is ubiquitious among mucinous ovarian tumors, in fact even this final case may have a cryptic RAS-activating alteration unseen by our hotspot screening strategy. Of particular importance, so-called “double hits” to this pathway were shown to overlap the same populations of cells in two cases where testing for this overlap was possible. This finding suggests different RAS mutations contribute, at least in part, unique functionality with respect to mucinous tumor progression.

Finally, the overall patterns of mutations amongst these tumors are not dissimilar to other mucinous tumor types, including pancreatic and appendiceal tumors [46–49]. Although extensive care was taken to exclude metastatic disease, limited certainty of primary ovarian tumor versus metastatic disease holds true for virtually all studies on MC and MBOT, and remains a concern here. However, an overlapping relationship, either with respect to the origins or mechanisms mediating transformation, between ovarian mucinous and other peritoneal mucinous tumors is not unrealistic. Commonalities between these mucinous cancers may help explain the inherent chemoresistance in contrast to other EOC’s and suggest so-called umbrella trial designs, grouping together cancers with similar molecular presentation, may provide a realistic option for treatment development in this relatively rare tumor type.

## Additional files

Additional file 1:
**Hotspot sequencing, cellularity estimates and HPV infection status data for 26 mucinous borderline tumors and 37 mucinous carcinomas.**

Additional file 2:
**p53 immunohistochemistry results and outcome data for entire cohort of MBOT and MC.**

Additional file 3:
**Survival Analysis for MC based on p53 immunohistochemistry.**

## Abbreviations

CISH: Chromogenic-*in situ* hybridization
COSMIC: Catalogue of somatic mutations in cancer
EOC: Epithelial ovarian cancer
FFPE: Formalin fixed paraffin embedded
FISH: Fluorescent-*in situ* hybridization
IHC: Immunohistochemistry
MAPK: Mitogen activated protein kinase
MBOT: Mucinous borderline tumor
MC: Mucinous carcinoma
