# Supplementary figures and images for: Targeted deep sequencing of mucinous ovarian tumors reveals multiple overlapping RAS-pathway activating mutations in borderline and cancerous neoplasms
Source: BMC Cancer. 2015 May 19;15:415. doi: 10.1186/s12885-015-1421-8 (PMC4494777; doi:10.1186/s12885-015-1421-8)

A

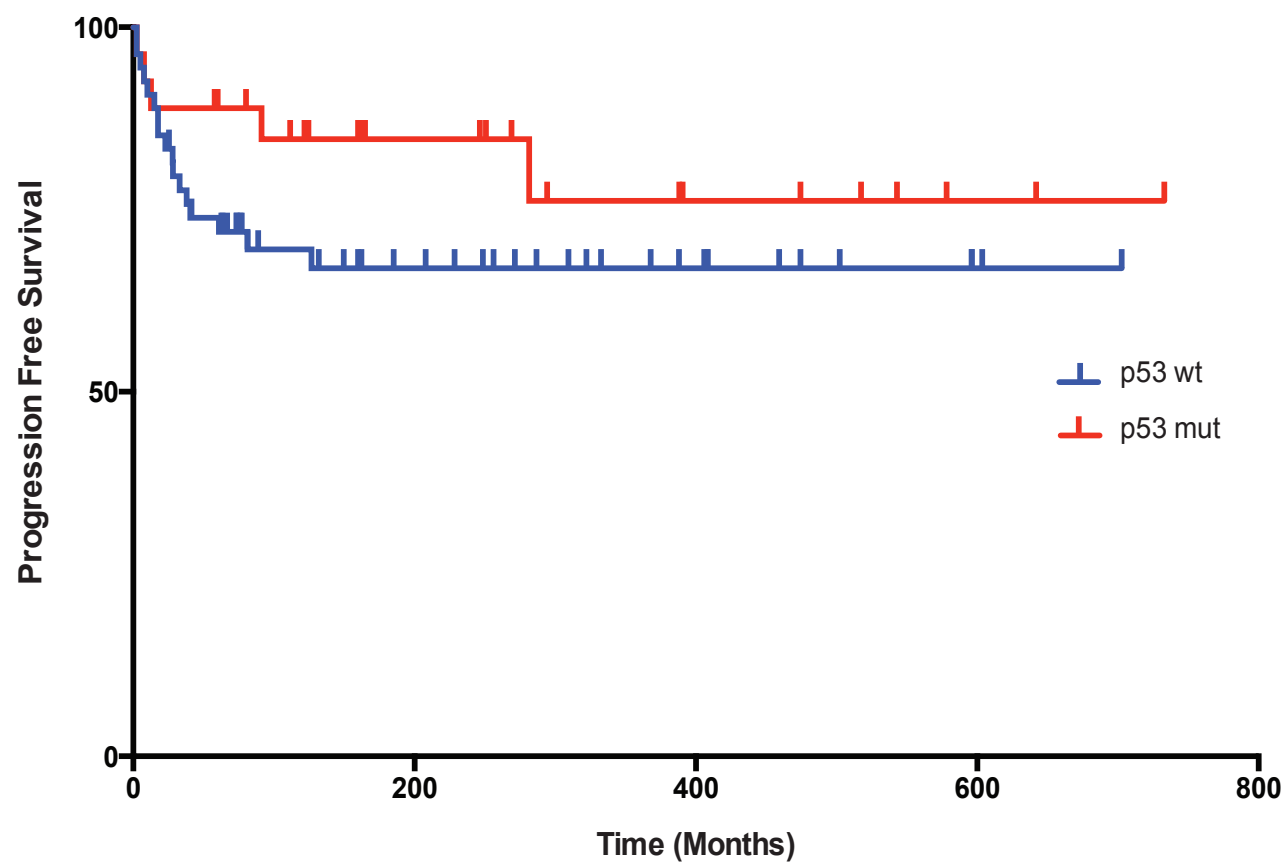

B

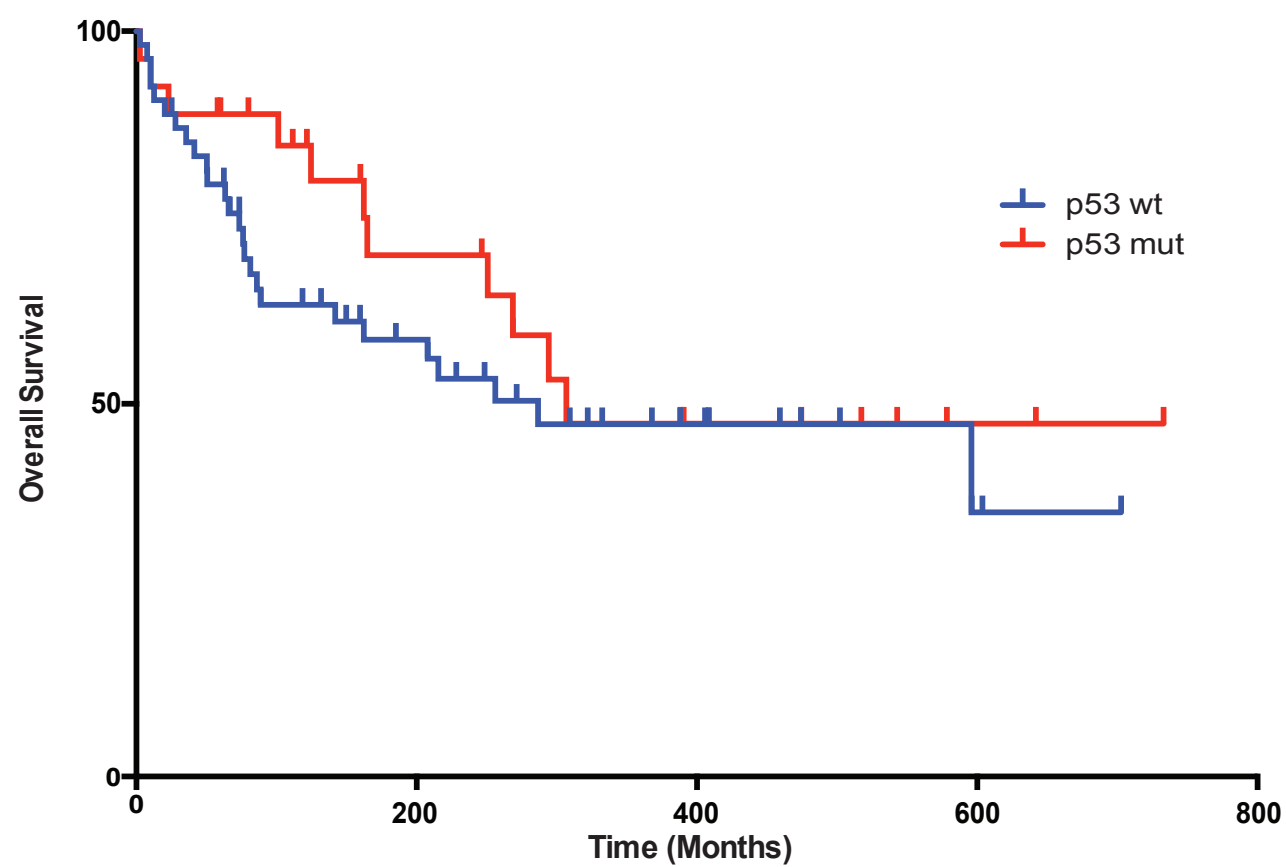

Supplement: Additional file 3: — Survival Analysis for MC based on p53 immunohistochemistry. [file 12885_2015_1421_MOESM3_ESM.pdf]
